# Recent advances of exosomal circRNAs in cancer and their potential clinical applications

**DOI:** 10.1186/s12967-023-04348-4

**Published:** 2023-07-31

**Authors:** Qian Yi, Jiaji Yue, Yang Liu, Houyin Shi, Wei Sun, Jianguo Feng, Weichao Sun

**Affiliations:** 1 Department of Physiology, School of Basic Medical Science, Southwest Medical University, Luzhou, 646000 China; 2 Department of Bone Joint and Bone Oncology, Shenzhen Second People’s Hospital, Shenzhen, 518035 Guangdong China; 3 Department of Anesthesiology, The Affiliated Hospital of Southwest Medical University, Luzhou, Sichuan China; 4 The Central Laboratory, Shenzhen Second People’s Hospital, Shenzhen, 518035 Guangdong China

**Keywords:** Exosome, circRNAs, Tumorigenesis, Disgnostic biomarker, Cancer treatment

## Abstract

Circular RNA (circRNA) is a type of non-coding RNA that forms a covalently closed, uninterrupted loop. The expression of circRNA differs among cell types and tissues, and various circRNAs are aberrantly expressed in a variety of diseases, including cancer. Aberrantly expressed circRNAs contribute to disease progression by acting as microRNA sponges, functional protein sponges, or novel templates for protein translation. Recent studies have shown that circRNAs are enriched in exosomes. Exosomes are spherical bilayer vesicles released by cells into extracellular spaces that mediate intercellular communication by delivering cargoes. These cargoes include metabolites, proteins, lipids, and RNA molecules. Exosome-mediated cell-cell or cell-microenvironment communications influence the progression of carcinogenesis by regulating cell proliferation, angiogenesis, metastasis as well as immune escape. In this review, we summarize the current knowledge about exosomal circRNAs in cancers and discuss their specific functions in tumorigenesis. Additionally, we discuss the potential value of exosomal circRNAs as diagnostic biomarkers and the potential applications of exosomal circRNA-based cancer therapy.

## Introduction

Cancer is a leading cause of death worldwide, particularly because of its high morbidity and mortality, and it has caused enormous pain to individuals, imposed a tremendous burden on families and health systems [1]. Conventional blood biomarkers are widely used for cancer diagnosis, but their low sensitivity and specificity limit their application. The early symptoms of many malignant tumors are not obvious, and most patients are diagnosed at an advanced stage of the disease [2, 3]. Therefore, it is crucial to explore new non-invasive biomarkers for the early diagnosis of malignant cancers. Currently, liquid biopsy was developed for detecting novel, highly accurate biomarkers in human body fluids [4, 5]. It is non-invasive, simpler, faster, and more accurate compared to traditional histological biopsy [6]. In addition, more dynamic monitoring of disease progression and recurrence is allowed through repeated sampling via liquid biopsy [7, 8].

Exosomes are spherical bilayer vesicles released by a variety of cells into extracellular spaces. They mediate the cell-cell or cell-environments’ communications by delivering cargoes, such as circular RNAs (circRNAs), microRNAs, mRNAs, DNAs, long non-coding RNAs (lncRNAs), proteins, and lipids [9, 10]. Exosomes are one of the main detection materials for liquid biopsy because they are present in almost all body fluids, including blood, saliva, urine, and cerebrospinal fluid [11]. CircRNA is a type of non-coding RNA with a covalently closed, uninterrupted loop [12]. Due to their special loop structure, circRNAs are relatively stable and not easily degraded when compared to linear RNAs [13]. Moreover, circRNAs are enriched in exosomes, and their expression remarkably changes under physiological or pathological conditions [14, 15]. These studies suggest that circRNAs in the exosomes of body fluids potentially represent novel biomarkers for monitoring cancer progression and predicting prognosis [16].

In this review, we summarize the biological functions of exosomal circRNAs and their significance in cancer progression. We also review the potential clinical applications of exosomal circRNAs as biomarkers in cancer diagnosis, disease judgement, and prognosis observation. In addition, we discuss the potential value of exosome-based circRNA delivery for targeted cancer treatment.

## Exosomes

**Fig. 1:**
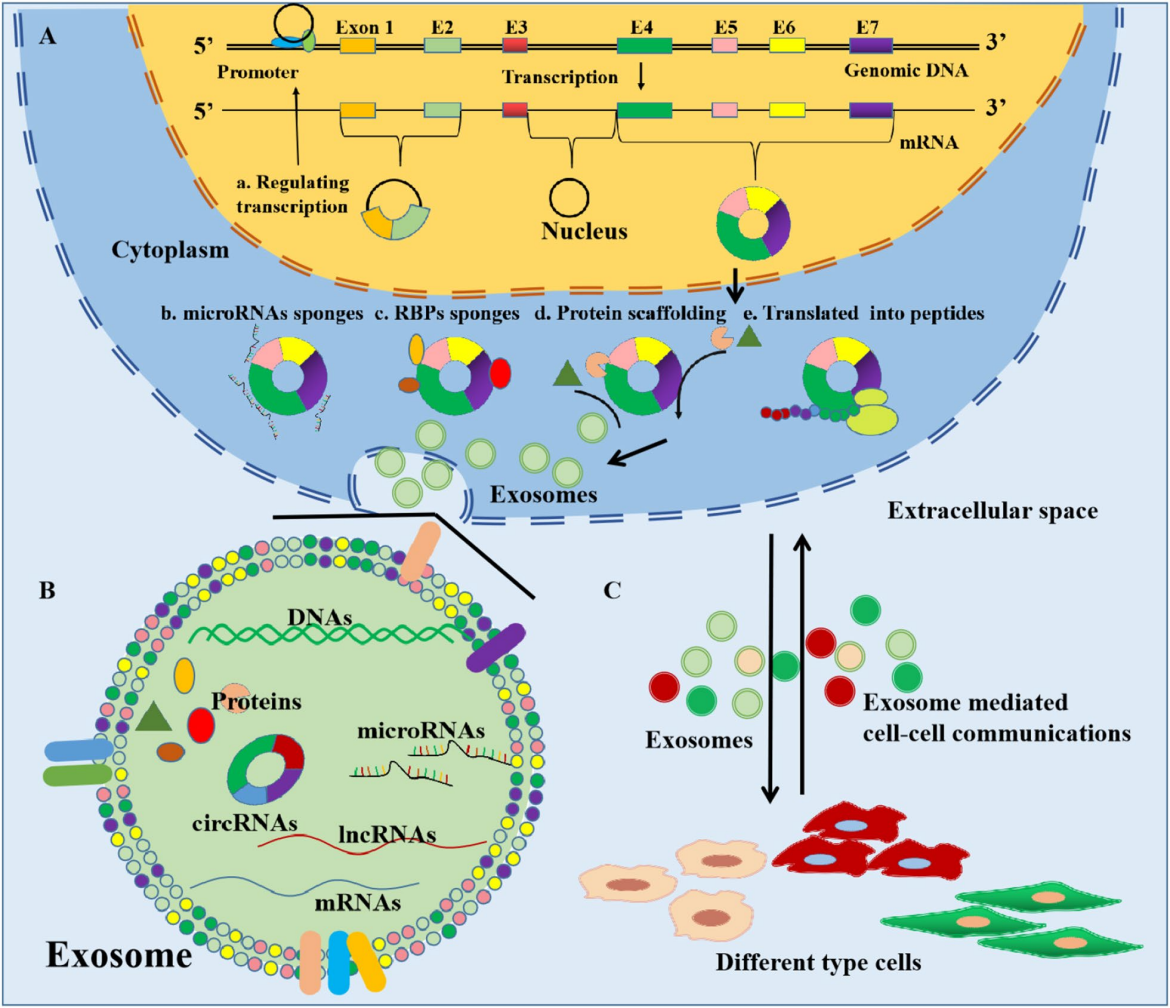
Structural schematic diagram of circRNA and exosomes. **A** The formation process of covalently closed, uninterrupted loop circRNAs and their biological functions; **B** the lipid bilayer structure of exosomes and its molecules contents; **C** Exosomes mediate the communications among different type cells

Exosome is one kind of extracellular vehicles with a spherical bilayer membrane structure and a diameter of approximately 50–150 nm [17] **(**Fig. 1**)**. Traditionally, exosomes are formed from endosomal compartment invaginations and are secreted from the plasma membrane [18]. It was found that almost all types of cells can normally secrete exosomes, which play a crucial role in regulating communication among cells, organs, tissues, and cellular microenvironments. Exosomes contain various molecular constituents, such as circRNAs, microRNAs, DNAs, long non-coding RNAs (lncRNA), proteins, lipids, and so on [19]. The special lipid bilayer structure of exosomes ensures that these contents cannot be degraded and can be easily absorbed by recipient cells [20]. Several studies have reported that the contents of exosomes change remarkably under pathological conditions and that cells can regulate each other’s biological processes via exosomes [21, 22]. For example, tumor-derived exosomes can contribute to angiogenesis and tumor metastasis by delivering these contents to human vascular endothelial cells [23]. Cancer-associated fibroblasts (CAFs) promote chemotherapy resistance of tumor cells via delivering microRNAs through exosomes [24]. CAFs-derived exosomal lncRNA H19 promotes the stemness and chemoresistance of colorectal cancer (CRC) [25]. Moreover, exosomes are widely present in body fluids including blood, saliva, urine, cerebrospinal fluid, and synovial fluid, implying that they could serve as primary detection materials for liquid biopsy [26, 27]. For example, Lydia et al. reported the role of exosomes and circulating miRNAs as a source of liquid biopsy biomarkers in ovarian cancer diagnosis [28]. Xiao et al. showed that circulating plasma exosomal lncRNAs could serve as prospective biomarkers in acute myeloid leukemia [29]. Exosomal circ-SCL38A1 can distinguish bladder cancer patients from healthy individuals, with a diagnostic accuracy of 0.878 [30]. These studies indicate that exosomes, especially exosomal RNA molecules, play an important role in cancer diagnosis and treatment.

## Biological functions of exosomal circRNAs in cancer

### General characteristics of circRNA

CircRNA is a type of non-coding RNA formed by back-splicing in which a downstream splice donor site is joined with an upstream splice acceptor site to form a covalently closed, uninterrupted loop [31, 32] (Fig. 1). It was first reported by Dr. Hsu, and it was thought to have no valuable biological functions [33]. However, some recent studies revealed that more than 180,000 circRNAs are present in human transcriptomes and that their expression is associated with both normal cellular biological processes and disease progression [34, 35]. Based on their origin, circRNAs are classified into three major types: circular intronic RNAs, exon-intron circRNAs and exonic circRNAs [12, 36]. CircRNA were confirmed to play multiple roles in the biological processes through acting microRNAs or RNA binding proteins sponges to regulate target gene expression, regulating gene transcription or splicing and acting as templates for protein translation [37–39]. Research has shown that dysregulated circRNAs are associated with the pathogenesis of many human diseases, particularly cancer. Such as, circRNAs has been reported contribute to cancer metastasis and immune escape [40, 41].

Recently, circRNAs were found to be localized to exosomes and capable of being transferred between cells via exosomes, thereby affecting tumor progression. For example, exosome-derived circ-TFDP2 promotes the proliferation of prostate cancer (PC) cells by inhibiting caspase-3-dependent cleavage of PARP1 and DNA damage [42]. Furthermore, Zhao et al. reported that exosome-mediated transfer of circ_0000338 enhances 5-fluorouracil resistance in CRC by regulating microRNA-217/485-3p [43]. Exosomal circ-GSE1 promoteS immune escape of hepatocellular carcinoma (HCC) by inducing the expansion of regulatory T cells via the regulation of miR-324-5p/TGFBR1/Smad3/Tregs axis [44]. Importantly, circRNAs have the potential to serve as biomarkers for cancer diagnosis due to their exosome localization and enrichment. Such as, exosomal circ_0004771 has been reported to be overexpressed in CRC, with area under the curve (AUC) values of 0.86 and 0.88 used to differentiate stage I/II CRC patients and CRC patients from healthy controls, respectively [45].

### Exosomal circRNAs and proliferation of cancer cells

Various exosomal circRNAs have been reported to regulate the proliferation of cancer cells. For example, exosomal circ-PDK1 promotes pancreatic cancer (PCa) cell proliferation by sponging miR-628-3p to activate the BPTF/c-Myc axis during hypoxia [46]. Furthermore, exosomal circ-PRRX1 promotes cell proliferation in vitro and tumor growth in vivo by sponging miR-596 and activating the NF-κB signaling pathway in gastric cancer (GC) [47]. According to a previous study, cancer-derived exosomal circ-SERPINE2 is shuttled to tumor-associated macrophages (TAMs), and it enhances IL-6 secretion, leading to increased proliferation of breast cancer cells [48]. TAM-secreted exosomal circ_0020256 promotes the proliferation and progression of cholangiocarcinoma by modulating the miR-432-5p/E2F3 axis [49]. In renal cell carcinoma (RCC), tumor-derived exosomal circ-PPKCI increases tumor cell proliferation via the miR-545-3p/CCND1 signaling pathway [50]. In HCC, adipocyte-derived exosomal circ-DB promotes tumor growth by suppressing miR-34a and activating the USP7/Cyclin A2 signaling pathway [51]. Furthermore, hepatic stellate cell-derived exosomal circ-WDR25 facilitates HCC cell proliferation by regulating the miR-4474-3p/ALOX15 axis [52]. Exosomal circ-RACGAP1 recruiteS PTBP1 to induce RIF1 deacetylation, which then activates the Wnt/β-catenin pathway and prmotes the proliferation of non-small cell lung cancer (NSCLC) cells [53]. Interesting, multiple myeloma (MM)-derived exosomal circ-HNRNPU encodes a novel 603-aa peptide, which regulates the bone marrow microenvironment and promotes cell proliferation [54].

However, Circ-LPAR1 expression in plasma exosomes was decreased in CRC and it suppressed the tumor cell proliferation by suppressing the translation of oncogene BRD4 [55]. Exosomal circ-PTPRA induced CRC cell cycle arrest and inhibited cell proliferation by enriching the level of SMAD4 via competitively binding to miR-671-5p [56]. Chen et al. reported that circ_0051443 was transmitted from normal cells to HCC cells via exosomes and suppressed the cell proliferation and malignant biological progression [57]. In oral squamous cell carcinoma (OSCC), exosomal circ-GDI2 was downregulated and its upregulation weakened the cell proliferation by regulating miR-424-5p/SCAI axis [58]. In addition, Chen et al. reported that tumor-suppressive circ-RHOBTB3 could be excreted out of CRC cells via exosomes and circ-RHOBTB3 suppressed cell growth and metastasis [59]. Besides, exosomal circ-BTG2 or circ_0004658 secreted from RBP-J overexpressed-macrophages inhibited glioma or HCC progression by regulating miR-25-3p/PTEN or miR-499b-5p/JAM3 pathway, respectively [60, 61].

### Exosomal circRNAs in metastasis

Exosomal circRNAs also have crucial function in regulating tumor metastasis. Circ-PACRGL is secreted by CRC cells, and acts as a miR-142-3p/ miR-506-3p sponge to activate the TGF-β-related signaling and promote metastasis [62]. In HCC, exosome-transmitted circMMP2 induced metastasis by sponging miR-136-5p and increasing MMP2 expression [63]. Moreover, exosomal circRAPGEF5 promoted the metastasis of lung adenocarcinoma through the miR-1236-3p/ZEB1 axis [64]. Tumor-derived exosomal circPSMA1 facilitated the metastasis in triple-negative breast cancer through the regulation of miR-637/Akt1/β-catenin regulatory axis [65]. Furthermore, exosomal circ_0081234 promoted the epithelial-mesenchymal transition (EMT) of PC cells [66]. Circ_0003028 induced EMT of HCC cells by exosome pathway via microRNA-498/ODC1 signaling [67]. And exosomal circ_007293 promoted EMT of papillary thyroid carcinoma cells via the regulation of the miR-653-5p/PAX6 axis [68]. In addition, the metastatic ability of HCC cells could be enhanced by transferring exosomal circRNA-100,338 to human umbilical vein endothelial cells (HUVECs), and promoting angiogenesis [69]. In GC, tumor-derived exosomal circ_0044366 promoted tube formation of HUVECs and enhanced cancer migration [70]. In ovarian cancer, exosomal circ-NFIX increased angiogenesis via miR-518a-3p/TRIM44/JAK/STAT1 pathway [71]. In esophageal squamous carcinoma, exosomal circ_0026611 contributed to LNM by interacting with N-α-acetyltransferase 10 (NAA10) to inhibit NAA10-mediated PROX1 acetylation [72].

However, Chen et al. reported that CAFs directly transferred circ-IFNGR2 into ovarian cancer cells and suppressed metastasis by activating miR-378/ST5 [73]. Moreover, bone marrow mesenchymal stem cell-derived exosomal circ_0006790 suppressed metastasis of pancreatic ductal adenocarcinoma by binding to CBX7 and regulating S100A11 DNA methylation [74]. Lin et al. found that exosomal circ_0072088 suppressed migration and invasion of hepatic carcinoma cells by regulating miR-375/MMP-16 [75]. In GC, the expression of exosomal circ-ITCH and circ-STAU2 were significantly downregulated, they suppressed the metastasis of GC by regulating miR-199a-5p/Klotho axis or miR-589/ CAPZA1 respectively [76, 77].

### Exosomal circRNAs in drug resistance

Exosomal circRNAs were associated with the drug resistance of cancers. Exosomal circ_0076305 promoted cisplatin (DDP) resistance of non-small cell lung cancer cell (NSCLC) by enhancing ABCC1 expression [78]. Circ-VMP1 and circ_0014235 were elevated in DDP-resistant NSCLC exosomes, they facilitated DPP resistance by regulating miR-524-5p/METTL3/SOX2 or miR-520a-5p/CDK4 axis, respectively [79]. In osteosarcoma, exosomal circ_103801 conferred DDP resistance by increasing the expression of MRP1 and p-glycoprotein [80]. Warburg effect promoted temozolomide (TMZ) resistant glioma cells releasing exosomal circ_0072083, which induced TMZ resistance of sensitive cells by regulating miR-1252-5p/NANOG [81]. Circ-ZNF91 was remarkably increased in exosomes of PCa under hypoxia condition and promoted gemcitabine resistance of normoxic PCa cells via regulating miR-23b-3p/SIRT1 and enhancing glycolysis [82]. In neuroblastoma, exosomal circ-DLGAP4 enhanced glycolysis and doxorubicin resistance via miR-143-HK2 axis [83]. Oxaliplatin-resistant CRC cells delivered exosomal circ_0005963 to sensitive cells, promoted drug resistance by miR-122 sponging and PKM2 upregulation [84]. Furthermore, exosomal circ_0091741 promoted oxaliplatin resistance of GC cells via the miR-330-3p/ TRIM14/Dvl2/Wnt/β-catenin pathway [85]. Exosomal circ-SFMBT2 and circ-XIAP were upregulated in docetaxel-resistant PC cells, their knockdown enhanced docetaxel sensitivity by regulating miR-136-5p/TRIB1 or miR-1182/TDP52 axis [86, 87]. Pan et al. reveled that exosomal circATG4B induced oxaliplatin resistance in CRC by encoding a novel protein to increase autophagy [88].

However, Xu et al. found that exosomal circ-FBXW7 led resistant cells sensitive to oxaliplatin and suppressed oxaliplatin efflux via sponging miR-18b-5p in CRC [89]. Moreover, circRNA-CREIT could be packaged into exosomes and disseminate doxorubicin sensitivity among TNBC cells by destabilizing PKR [90]. In liver cancer, transarterial chemoembolization increased the expression of exosomal circ-G004213, which promoted DDP sensitivity by regulating miR-513b-5p/PRPF39 axis [91].

We summarized exosomal circRNAs and their function in tumorigenesis in Table 1.

**Table 1:** Exosomal circRNAs and their function in tumorigenesis

Tumor type	circRNA	Target molecules	Function	References
NSCLC	Circ-RACGAP1	Wnt/β-catenin	Proliferation	[53]
NSCLC	Circ_0076305	miR-186-5p/ABCC1	DDP resistance	[78]
NSCLC	Circ-VMP1 Circ_0014235	miR-524-5p/SOX2 miR-520a-5p/CDK4	DDP resistance	[79]
NSCLC	Circ-STAB2	miR-330-5p/PEAK1	Progression	[92, 93]
NSCLC	Circ_0007385	miR-1253/FAM83A	Proliferation, stemness	[94]
NSCLC	Circ_0008717	miR-1287-5p/PAK2	Tumorigenicity	[95]
NSCLC	Circ-ARHGAP10	miR-638/FAM83F	Progression	[96]
NSCLC	Circ_102481	miR-30a-5p/ROR1	EGFR-TKIs resistance	[97]
NSCLC	Circ-PLK1	miR-1294/HMGA1	Progression	[98]
NSCLC	Circ_0014235	miR-520a-5p/CDK4	DDP resistance	[99]
NSCLC	Circ_0002130	miR-498	Osimertinib resistance	[100]
NSCLC	Circ-CCDC134	miR-625-5p/NFAT5	Progression	[101]
Lung cancer	Circ-DNER	miR-139-5p/ITGB8	Paclitaxel resistance	[102]
LUAD	CircRAPGEF5	miR-1236-3p/ZEB1	Metastasis	[64]
CRC	Circ-PACRGL	miR-142-3p/miR-506-3p	Metastasis	[62]
CRC	Circ_0005963	miR-122	Oxaliplatin resistance	[84]
CRC	CircATG4B	Autophagy	Oxaliplatin resistance	[88]
CRC	Circ_0007334	miR/KLF12	Progression	[103]
CRC	Circ-COG2	miR-1305/TGF-β2/smad3	Progression	[104]
CRC	Circ-FMN2	miR-338-3p/MSI1	Progression	[105]
CRC	CircCOL1A2	miR-665/LASP1	Progression	[106]
CRC	Circ_0005615	miR-873-5p/FOSL2	Progression	[107]
CRC	Circ_0000395	miR-432-5p/MYH9	Progression	[108]
CRC	Circ-TUBGCP4	miR-146b-3p/PDK/Akt	Metastasis	[109]
CRC	Circ-PABPC1	miR-874/microRNA-1929	Metastasis	[110]
CRC	Circ-133a	miR-133a/GEF-H1/RhoA	Metastasis	[111]
HCC	Circ-DB	miR-34a/USP7/Cyclin A2	Proliferation	[51]
HCC	Circ-WDR25	miR-4474-3p/ALOX15	Proliferation	[52]
HCC	CircMMP2	miR-136-5p/MMP2	Metastasis	[63]
HCC	Circ_0003028	miR-498/ODC1	EMT process	[67]
HCC	Circ_100338	Angiogenesis	Metastasis	[69]
HCC	Circ-Cdr1as	miR-1270	Progression	[112]
HCC	Circ-TTLL5	miR-136-5p/KIAA1522	Metastasis	[113]
HCC	Circ-SORE	YBX1	Sorafenib resistance	[114]
HCC	Circ-PAK1	YAP	Lenvatinib resistance	[115]
HCC	Circ-ZFR	STAT3/NF-κB pathway	DDP resistance	[116]
Breast cancer	Circ-SERPINE2	/	Proliferation	[48]
Breast cancer	CircPSMA1	miR-637/Akt1/β-catenin	Metastasis	[65]
Breast cancer	Circ-MMP11	miR-153-3P/ANLN	Lapatinib resistance	[117]
Breast cancer	CCirc-UBE2D2	miR-200a-3p	Tamoxifen resistance	[118]
Breast cancer	Circ-CARM1	miR-1252-5p/PFKFB2	Glycolysis, progression	[119]
Breast cancer	Circ-EGFR	miR-1299/EGFR	Pirarubicin resistance	[120]
Gastric cancer	Circ-PRRX1	miR-596	Proliferation	[47]
Gastric cancer	Circ_0044366	/	Metastasis	[70]
Gastric cancer	Circ_0091741	miR-330-3p/ TRIM14	Oxaliplatin resistance	[85]
Gastric cancer	Circ-NRIP1	miR-145-5p/AKT1/mTOR	Metastasis	[121]
Gastric cancer	Circ_0001789	miR-140-3p/PAK2	Progression	[122]
Gastric cancer	Circ_0063562	miR-449a/SHMT2	DDP resistance	[123]
Gastric cancer	Circ-PVT1	miR-301-5p/YAP1	DDP resistance	[124]
Gastric cancer	Circ-LDLRAD3	miR-588/SOX5	DDP resistance	[125]
Gastric cancer	Circ_0032821	miR-515-5p/SOX9	Oxaliplatin resistance	[126]
Glioma	Circ_0072083	miR-1252-5p/NANOG	TMZ resistance	[81]
Glioma	Circ-WDR62	miR-370-3p/MGMT	TMZ resistance	[127]
Glioma	Circ-GLS3	miR − 548 m/MED31	TMZ resistance	[128]
Glioma	Circ_0043949	miR-876-3p/ITGA1	TMZ resistance	[129]
Glioblastoma	Circ-AHCY	miR-1294/ Wnt/β-catenin	Proliferation	[130]
Glioblastoma	Circ_0012381	miR-340-5p/CCL2/CCR2	Proliferation	[131]
Glioblastoma	Circ-KIF18A	FOXC2/PI3K/AKT	Angiogenesis	[132]
Prostate cancer	Circ_0081234	/	EMT process	[66]
Prostate cancer	Circ-SFMBT2	miR-136-5p/TRIB1	Docetaxel resistance	[86]
Prostate cancer	Circ-XIAP	miR-1182/TDP52	Docetaxel resistance	[87]
Prostate cancer	Circ-KDM4A	miR-338-3p/CUL4B	Malignancy	[133]
Ovarian cancer	Circ-NFIX	miR-518a-3p/TRIM44	Angiogenesis	[71]
Ovarian cancer	Circ-PIP5K1A	miR-942/NFIB	DDP resistance	[134]
Ovarian cancer	Circ-Foxp1	miR-22/miR-150-3p	DDP resistance	[135]
Ovarian cancer	Circ_0007841	miR-532-5p/NFIB	DDP resistance	[136]
PCa	Circ-PDK1	miR-628-3p/BPTF/c-Myc	Proliferation	[46]
PCa	Circ-ZNF91	miR-23b-3p/SIRT1	Gemcitabine resistance	[82]
PCa	Circ-IARS	miR-122	Metastasis	[137]
EC	Circ_0000337	miR-337-3p	DDP resistance	[138]
CCA	Circ_0020256	miR-432-5p/E2F3	Proliferation	[49]
RCC	Circ-PRKCI	miR-545-3p/CCND1	Proliferation	[50]
MM	Circ-HNRNPU	/	Proliferation	[54]
PTC	Circ_007293	miR-653-5p/PAX6	EMT process	[68]
ESCC	Circ_0026611	/	LNM	[72]
Osteosarcoma	Circ_103801	/	DDP resistance	[80]
Neuroblastoma	Circ-DLGAP4	miR-143-HK2	Doxorubicin resistance	[83]
Cervical cancer	Circ_0074269	miR-485-5p/TUFT1	DDP resistance	[139]
Melanoma	Circ_0001005	miRs sponges	Vemurafenib resistance	[140]
NPC	Circ-PARD3	miR-579-3p/SIRT1	Cisplatin resistance	[141]
CCA	Circ-CCAC1	EZH2	Angiogenesis	[142]

### Exosomal circRNAs in tumor immunity

**Fig. 2:**
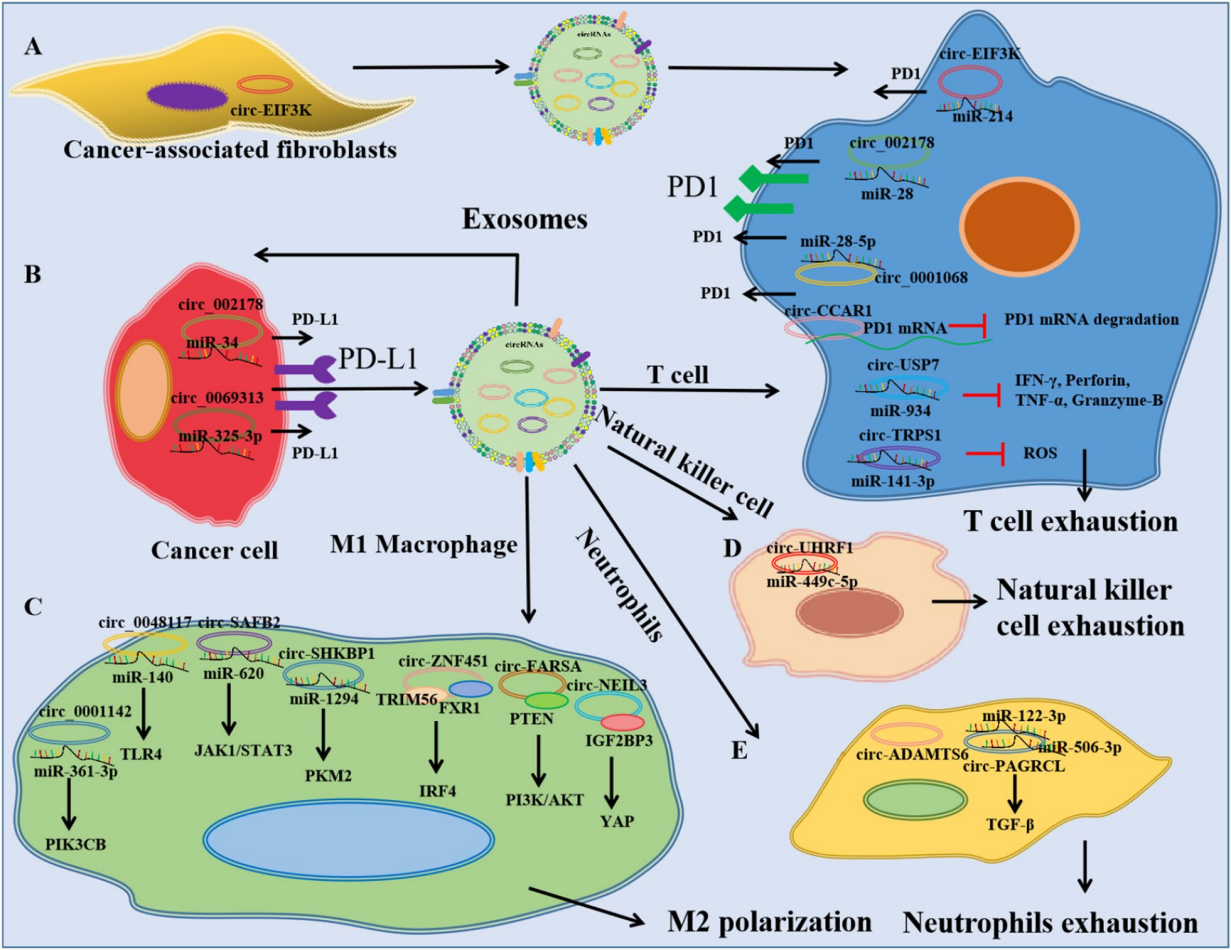
Exosomal circRNAs mediate the communication between tumor cells and immune cells. **A** The effect of cancer-associated fibroblasts-derived exosomal circRNAs on T cells; **B** The effect of cancer cell-derived exosomal circRNAs on tumor cells; **C** The effect of cancer cell-derived exosomal circRNAs on Macrophage cells; **D** The effect of cancer cell-derived exosomal circRNAs on Natural killer cells; **E**: The effect of cancer cell-derived exosomal circRNAs on Neutrophils

Exosomal circRNAs mediate the communication between tumor cells and immune cells (Fig. 2). In bladder cancer, exosome-derived circ-TRPS1 promotes CD8 + T cell exhaustion and the malignant phenotype by sponging miR-141-3p [143]. In NSCLC, upregulated plasma exosomal circ-USP7 inhibites CD8 + T cell function by sponging miR-934 and increasing SHP2 expression [144]. In LUAD, exosomal circ_002178 can be delivered to CD8 + T cells to induce PD1 expression and T cell exhaustion [145]. In ovarian cancer, exosomal circ-0001068 can be delivered to T cells and induced PD1 expression by sponging miR-28-5p [146]. In HCC, exosomal circ-CCAR1 promotes CD8 + T cell dysfunction by stabilizing the PD1 protein [147]. In OSCC, the transfer of circ_0069313 to Treg cells promotes immune escape by inhibiting miR-325-3p-induced Foxp3 degradation [148]. Moreover, CAF-derived exosomal circ-EIF3K increases the PD-L1 expression in CRC [149].

In NSCLC, exosomal circ-SHKBP1 or circ-FARSA promotes M2 polarization and cancer progression via the miR-1294/PKM2 or PTEN/PI3K/AKT pathway [150, 151]. In glioma, exosomal circ-NEL3 induces macrophage immunosuppressive polarization by stabilizing the oncogenic protein IGF2BP3 [152]. In LUAD, exosomal circ-ZNF451 restrains anti-PD1 treatment by polarizing macrophages and complexing with TRIM56 and FXR1 [153]. In breast cancer, exosomal circ_0001142 is released by cancer cells under endoplasmic reticulum stress, and it induces M2 polarization of macrophages [154]. In RCC, exosomal circ-SAFB2 reshapes the tumor environment, mediates M2 macrophage polarization, and promotes tumor progression [155]. In esophageal squamous cell carcinoma, tumor-derived exosomal circ_0048117 facilitates M2 macrophage polarization by regulating microRNA-140/TLR4 axis [156].

In HCC, cancer cells secrete exosomal circ-UHRF1, which induces natural killer cell exhaustion and promotes immune therapy resistance by regulating the miR-449c-5p/TIM3 axis [157]. CRC-derived exosomal circ-PACRGL regulates the differentiation of N1/N2 neutrophils [62]. Wang et al. reported that upregulated expression of plasma exosomal circ-ADAMTS6 is positively related to neutrophil extracellular traps in cholangiocarcinoma [158].

## Potential clinical applications of exosomal circRNAs

### Exosomal circRNAs in cancer diagnosis

CircRNAs have a special stable tertiary structure, and it has been reported that their expression is not significantly altered after 24 h of incubation at room temperature [14]. Furthermore, circRNAs were found to be dysregulated under pathological conditions and enriched in exosomes, which could be detected in body fluids such as blood, serum, urine, saliva, and cerebrospinal fluid [14, 15]. These features indicate that exosomal circRNAs can serve as biomarkers for cancer diagnosis. Xu et al. found that the expression of circ_0109046 and circ_0002577 were higher in exosomes isolated from serum samples of patients with stage III endometrial adenocarcinoma compared to healthy controls [159]. Xu et al. reported that circ-SHKBP1 is a promising circulating biomarker for GC diagnosis and prognosis due to its upregulation in serum and positive relationship with advanced TNM stage and poor survival [160]. Deng et al. reported that oral squamous cell carcinoma patients with higher expression of exosomal circ_047733 showed a lower risk of LNM [161]. Plasma exosome-derived circ_0055202, circ_0074920, and circ_0043722 are upregulated in glioblastoma multiforme and associated with tumor progression [162]. Furthermore, Hong et al. revealed that circ_0006220 and circ_0001666 are highly expressed in exosomes in the plasma of PCa patients compared to healthy controls and that they are associated LNM and tumor size. The AUC values were 0.7817 for circ_0006220, 0.8062 for circ_0001666, and 0.884 for the combined diagnosis [163]. The expressions of circ_0001492, circ_0001439, and circ_0000896 were significantly higher in the serum exosomes of LUAD patients, and the combination of these exosomal circRNAs had diagnostic sensitivity and specificity with an AUC value of 0.805 [164]. Furthermore, circ_0028861 was identified as a novel biomarker for HCC diagnosis, with an AUC of 0.79, and was capable of detecting small (AUC = 0.81), early-stage (AUC = 0.82), and AFP-negative (AUC = 0.78) tumors [165]. What’s more, exosomal circ_0015286 has an oncogenic function in GC, and its expression is closely associated with tumor size, TNM stage, LNM, and overall survival of GC patients [166]. Besides, clinical data have shown that exosomal circ_0000437 is enriched in the serum of GC patients and associated with LNM [167]. In addition, Wang et al. identified circ-SLC38A1 in the serum exosomes of bladder cancer patients, which could distinguish bladder cancer patients from healthy individuals with a diagnostic accuracy of 0.878 [30].

Other exosomal circRNAs that could serve as potential biomarkers for cancer diagnosis are summarized in Table 2.

**Table 2:** Exosomal circRNAs in body fluids for cancer diagnosis

Cancer	circRNAs	Level	Function	References
CRC	Circ-LPAR1	Down	Diagnostic biomarker (AUC 0.875)	[55]
CRC	Circ-GAPVD1	Up	Diagnostic biomarker (AUC 0.7662)	[168]
CRC	Circ-HIPK3	Up	Diagnostic biomarker (AUC 0.771)	[169]
CRC	Circ-PNN	Up	Early-stage diagnosis (AUC 0.854)	[170]
GC	Circ_0015286	Up	Diagnostic biomarker	[166]
GC	Circ_0000437	Up	Associated with LNM	[167]
GC	Circ-CDR1as	Up	Diagnostic biomarker (AUC 0.536)	[171]
GC	Circ_0065149	Down	Early diagnosis and prognosis prediction (AUC 0.64)	[172]
GC	Circ-KIA1244	Down	TNM stage and lymphatic metastasis (AUC 0.7481)	[173]
GC	Circ_0000419	Down	Diagnostic biomarker (AUC 0.84)	[174]
BC	Circ-MMP11	Up	Diagnostic biomarker (AUC 0.9444)	[117]
BC	Circ-HIF1A	Up	Diagnostic biomarker (AUC 0.897)	[175]
BC	Circ_0000615	Up	Diagnostic biomarker (AUC 0.904)	[176]
NSCLC	Circ_0047921, Circ_0056285, Circ_0007761	-	Diagnostic biomarker in the Chinese population (AUC 0.89, 0.820)	[177]
NSCLC	Circ_0048856	Up	Diagnostic biomarker (AUC 0.943)	[178]
NSCLC	Circ_0069313	Up	Diagnostic biomarker (AUC 0.749)	[179]
NSCLC	Circ-ERBB2IP	Up	Positively correlated with malignant (AUC 0.9168)	[180]
LUAD	Circ_0001492, Circ_0001439, Circ_0000896	Up	Diagnostic biomarker (AUC 0.805)	[164]
LUAD	Circ_0056616	Up	Biomarker for lymph node metastasis (AUC 0.812)	[181]
LUAD	Circ_0013958	Up	TNM stage and lymphatic metastasis (AUC 0.815)	[182]
LUSC	Circ_0014235, Circ_0025580	Up	Diagnostic biomarker (AUC 0.8)	[183]
Lung cancer	Circ_0002490, Circ_0087357, Circ_0004891, Circ_0074368	Down	Diagnostic biomarker (AUC 0.833, 0.793, 0.773, 0.730)	[184]
HCC	Circ_0051443	Down	Diagnostic biomarker (AUC 0.8089)	[57]
HCC	Circ_0028861	Down	Diagnostic biomarker	[165]
HCC	Circ-SMARCA5	Down	Diagnostic biomarker (AUC 0.862)	[185]
HCC	Circ_0006602	Up	Diagnostic biomarker (AUC 0.907)	[186]
HCC	Circ_0004001, Circ_0004123, Circ_0075792	–	Positively correlated with the TNM stage and tumor size	[187]
ESCC	Circ_0026611	Up	Lymph node-metastatic biomarker (AUC 0.724)	[188]
ESCC	Circ_0001946	Up	Predict the recurrence and prognosis (AUC 0.894)	[189]
MM	Circ-MYC	Up	Recurrence and Bortezomib resistance (AUC 0.924)	[190]
Ovarian	Circ_0001068	Up	Diagnostic biomarker (AUC 0.9697)	[146]
AC	Circ_0109049 Circ_0002577	Up	Diagnostic stage III biomarker	[159]
OSCC	Circ_047733	Down	Negatively with LNM	[161]
GBM	Circ_0055202, Circ_0074920, Circ_0043722	Up	Predict the tumor progression	[162]
PCa	Circ_0006220 Circ_0001666	Up	Diagnostic biomarker (AUC 0.884)	[163]

### Exosome-based circRNA delivery for cancer therapy

Exosomes can transport RNA molecules and deliver therapeutic drugs to cancer cells with good histocompatibility, high efficiency, and low cytotoxicity. Researchers have reported that some circRNAs have tumor suppressor functions, and the therapeutic delivery of exosomal circRNAs could suppress the proliferation, metastasis, drug resistance and progression of malignant tumors. Circ-EPB41L2 is downregulated in the exosomes of CRC patients, and exosome-mediated circ-EPB41L2 suppresses tumor progression by regulating the PTEN/AKT signaling pathway [191]. Zhang et al. reported that exosome-delivered circ-STAU2 inhibites the progression of GC by targeting the miR-589/CAPZA1 axis [77]. Moreover, Sang et al. reported that the exosomal transmission of circ-RELL1 suppresses the proliferation, invasion, and migration of GC cells [192]. Circ-DIDO1 is downregulated in GC, and circ-loaded, RGD-modified engineering exosomes significantly inhibit the proliferation, migration, and invasion of GC cells both in vivo and in vitro [193]. Furthermore, Circ-CREIT is aberrantly downregulated in doxorubicin-resistant TNBC cells and is associated with a poor prognosis. The exosomal transmission of circ-CREIT could disseminate doxorubicin sensitivity among these cells by destabilizing PKR [90]. Circ_0094343 is significantly downregulated in CRC, and exosome-carried circ_0094343 playes a tumor suppressor role and improves the chemosensitivity of tumor cells to 5-fluorouracil, oxaliplatin and doxorubicin [194].

Tumor microenvironment-associated cells also play tumor suppressor roles by delivering exosomal circRNAs to cancer cells. For example, CAF-derived exosomes deliver circ-IFNGR2 to ovarian cancer cells and inhibit malignant tumor progression by regulating the microRNA-378/ST5 axis [73]. Moreover, RBP-J-overexpressed- macrophage-derived exosomal circ-BTG2 or circ_0004658 inhibit glioma or HCC progression [60, 61]. Furthermore, Yao et al. reported that exosomal circ_0030167 derived from bone marrow-derived mesenchymal stem cells (BM-MSCs) exhibit significant tumor suppressor function in PCa by sponging microRNA-338-3p and targeting the Wif1/Wnt8/β-catenin axis [195]. BM-MSC-derived exosomal circ_0006790 inhibits growth, metastasis, and immune escape in pancreatic ductal adenocarcinoma [74].

Besides, Nanoparticles or exosomes mediated circRNAs silencing also a potential strategy for cancer treatment. For example, nanoparticles delivery si-circ-ROBO1 to hepatocellular carcinoma cells circ-ROBO1 inhibited tumor progression by modulating circ-ROBO1/miR-130a-5p/CCNT2 Axis[196]. And natural compound matrine blocked circ-SLC7A6 exosome secretion from CAFs, and then inhibited CRC cell proliferation and invasion[197]. These studies indicate that exosomal delivery of tumor-suppressing circRNAs or exosomal circRNAs-based engineering of exosomes or exosome circRNAs release inhibition may be novel cancer therapies.

The recent data reporter about “exosome-based circRNA delivery for cancer therapy” were summarized in Table 3.

**Table 3:** Exosome-based circRNA delivery for cancer therapy

Cancer	circRNAs	Source	Function	References
SCLC	Circ-SH3PXD2A	Circ-SH3PXD2A-overexpressing cells	Decreased chemoresistance and cell proliferation	[198]
Lung	Circ-RABL2B	Circ-RABL2B-overexpressing cells	Impoverished stemness, and promoted erlotinib sensitivity	[199]
CRC	Circ-PTPRA	Circ-PTPRA transfected cells	Inhibited tumorigenesis and promoted radiosensitivity	[56]
CRC	Circ-RHOBTB3	ASOs treated CRC	Inhibited CRC growth and metastasis	[59]
CRC	Circ-FBXW7	circ-FBXW7-transfected FHC cells	Ameliorated chemoresistance to oxaliplatin	[89]
CRC	Circ-EPB41L2	Circ-EPB41L2 transfected cells	Inhibited proliferation and metastasis	[191]
CRC	Circ_0094343	NCM460	Improved chemosensitivity	[194]
HCC	Circ_0051443	HL-7702 cell	Suppressed tumor progression	[57]
HCC	Circ_0004658	RBP-J-overexpressed- macrophage	Inhibited the progression	[61]
HCC	Circ_0072088	HCC cells	Suppressed the metastasis	[75]
HCC	Circ-G004213	/	Promoted cisplatin sensitivity	[91]
PDAC	Circ_0006790	BMSC	Inhibited growth, metastasis, and immune escape	[74]
PDAC	Circ_0012634	Pancreatic ductal epithelial cel1l	Restrained PDAC progression	[200]
Gastric	Circ-ITCH	Circ-ITCH-transfected cells	Suppressed the metastasis	[76]
Gastric	CircSTAU2	GES-1 cells	Inhibited the progression	[77]
Gastric	Circ_0017252	GC cells	Inhibited macrophage M2 polarization	[201]
Gastric	Circ-RELL1	/	Suppressed the malignant behavior	[192]
Gastric	Circ-DIDO1	Circ-DIDO1 transfected 293T	Suppressed tumor progression	[193]
Glioma	Circ-BTG2	RBP-J-overexpressed- macrophage	Inhibited the progression	[60]
Ovarian	CircIFNGR2	CAF	Inhibited the malignant progression	[73]
PCa	Circ_0030167	BMSCs	Inhibited the stemness	[195]
TNBC	Circ-CREIT	/	Overcome doxorubicin resistance	[90]
OSCC	Cicr-GDI2	Circ-GDI2-transfected CAL27 cells	Suppressed tumor progression	[58]
RCC	Circ-SPIRE1	Circ-SPIRE1 over-expressed cells	Suppressed angiogenesis and metastasis	[202]
NPC	Circ-FIP1L1	Guggulsterone treated HNE1 cells	Repressed HUVECs angiogenesis	[203]

## Discussion and conclusion

In this review, we comprehensively summarized current knowledge about the crucial function of exosomal circRNAs in tumor cell proliferation, metastasis, drug resistance, and progression. Several studies have mainly focused their research on tumor-derived exosomal circRNAs, but cancer cells exist in a complex and comprehensive microenvironment, and tumor progression involves the participation of various types of cells. Further research needs to focus on the role of exosomal circRNAs that derived from CAF, TAM, and other immune cells in tumor initiation, development, and progression.

Although numerous studies have revealed the abundance and diverse contributions of exosomal circRNAs to tumorigenesis, many questions remain unanswered. CircRNAs are mainly synthesized and retained in the nucleus, and the regulatory mechanisms of exosomes localization of circRNAs are not fully understood. A recent study reported that N6-methyladenosine modification facilitates the cytoplasmic export of circRNAs [204], indicating that m6A modification may regulate the exosome sorting of circRNAs. Moreover, it has been reported that some RNA-binding proteins, such as Argonaute and mannose-binding lectin can bind to circRNAs [205], and exosome sorting of microRNAs is dependent on the ESCRT complex, with Ago2 being the critical protein [206], indicating that exosome-associated RBPs may regulate the exosome sorting of circRNAs. In addition, hnRNPA2B1 mediates the exosome sorting of circ-NEIL3 and circ-CCAR1 [147, 152]. Additional studies are needed to illustrate the regulatory mechanisms of exosomes localization of circRNAs.

Currently, a large number of studies have proved that exosomal circRNAs have a potential value in cancer diagnosis and prognosis observation due to their highly conserved structure and tissue-specific expression patterns. More experimental verification, larger cohorts, and sufficient theoretical results are warranted to prove the clinical applicable of exosomal circRNAs as biomarkers. Besides, research into engineered exosomes as an approach for targeted cancer treatment is still in its infancy, future efforts should focus on identifying specific exosomal circRNAs and developing efficient and safe engineered exosomes for clinical application.

In conclusion, we comprehensively reviewed current knowledge about the crucial function of exosomal circRNAs in cancer progression, discussed their potential value in cancer diagnosis and prognosis observation, and described the potential utility of engineered exosomes for targeted cancer treatment.

## Data Availability

Not applicable.

## Abbreviations

circRNA: Circular RNA
lncRNAs: Long non-coding RNAs
AUC: Area under the curve
CAFs: Cancer-associated fibroblasts
CRC: Colorectal cancer
EC: Esophageal cancer
BC: Breast cancer
PC: Prostate cancer
HCC: Hepatocellular carcinoma
PCa: Pancreatic cancer
GC: Gastric cancer
TAMs: Tumor-associated macrophages
RCC: Renal cell carcinoma
NSCLC: Non-small cell lung cancer
SCLC: Small cell lung cancer
MM: Multiple myeloma
EMT: Epithelial-mesenchymal transition
HUVECs: Human umbilical vein endothelial cells
NAA10: N-α-acetyltransferase 10
TMZ: Temozolomide
BM-MSCs: Bone marrow-derived mesenchymal stem cells
PDAC: Pancreatic ductal adenocarcinoma
NPC: Nasopharyngeal carcinoma
CCA: Cholangiocarcinoma
AC: Endometrial adenocarcinoma
OSCC: Oral squamous cell carcinoma
GBM: Glioblastoma multiforme
PTC: Papillary thyroid carcinoma
ESCC: Esophageal squamous carcinoma
CCA: Cholangiocarcinoma
